# Early postoperative outcomes of staging laparoscopy for peritoneal metastases with or without pressurized intra-peritoneal aerosol chemotherapy (PIPAC)

**DOI:** 10.1186/s12893-022-01572-5

**Published:** 2022-03-30

**Authors:** Hugo Teixeira Farinha, Daphné Mattille, Styliani Mantziari, Nicolas Demartines, Martin Hübner

**Affiliations:** Department of Visceral Surgery, Lausanne University Hospital (CHUV), University of Lausanne (UNIL), Rue du Bugnon, 46, 1005 Lausanne, Switzerland

**Keywords:** PIPAC, Peritoneal metastases, Peritoneal Cancer Index, Staging laparoscopy

## Abstract

**Background:**

Pressurized intraperitoneal aerosol chemotherapy (PIPAC) has been introduced for palliative treatment of peritoneal surface malignancies (PSM) and is currently tested also in the neoadjuvant and prophylactic setting. The aim was therefore to compare safety and tolerance of staging laparoscopy with or without PIPAC.

**Methods:**

This retrospective analysis compared consecutive patients undergoing staging laparoscopy alone for oesogastric cancer with patients having PIPAC for suspected PSM of various origins from January 2015 until January 2020. Safety was assessed by use of the Clavien classification for complications and CTCAE for capturing of adverse events. Pain and nausea were documented by use of a visual analogue scale (VAS: 0–10: maximal intensity).

**Results:**

Overall, 25 PIPAC procedures were compared to 24 staging laparoscopies. PIPAC procedures took a median of 35 min (IQR: 25–67) longer. Four patients experienced at least one complication in either group (p = 0.741). No differences were noted for postoperative nausea (p = 0.961) and pain levels (p = 0.156). Median hospital stay was 2 (IQR: 1–3) for PIPAC and 1 (IQR: 1–2) for the laparoscopy group (p = 0.104).

**Conclusions:**

The addition of PIPAC did not jeopardize safety and postoperative outcomes of staging laparoscopy alone. Further studies need to clarify its oncological benefits.

## Background

Treatment of peritoneal metastases (PM) remains an oncological and surgical challenge [1–4]. Pressurized intraperitoneal aerosol chemotherapy (PIPAC) has been proposed as a novel method of intraperitoneal drug delivery for patients with peritoneal surface malignancies (PSM), claiming improved distribution, enhanced tissue uptake, better tolerance and repeatability using minimally invasive access [5, 6]. Recent systematic reviews confirmed PIPAC to be a safe and promising treatment option for patients with unresectable advanced isolated peritoneal disease, refractory to systemic treatment [7, 8]. Objective clinical response was reported in the palliative setting in 62–88% of patients with ovarian cancer, between 50 and 91% for gastric cancer and 71–86% for colorectal cancer [8]. PIPAC combined with systemic chemotherapy was also recently suggested as neoadjuvant treatment, in an attempt to make initially non-resectable patients eligible for secondary CRS and HIPEC with curative intent [9, 10]. Hence, it appears reasonable to consider PIPAC in the neoadjuvant and prophylactic setting while performing the initial staging laparoscopy in patients at high risk for presence of microscopic deposits or development of metachronous PSM [11].

The aim of this study was to assess safety and tolerance of the addition of PIPAC to baseline staging laparoscopy, for patients with high-risk features for PSM.

## Methods

This single centre retrospective comparative study included all consecutive patients admitted for staging laparoscopy, during workup of intra-abdominal neoplasia of various origin (colorectal, appendicular, gastric and ovarian). Indications for adding PIPAC were either suspected PSM on baseline imaging or a high-risk constellation. Indications for the procedure were decided in the multidisciplinary tumor board and all patients signed informed consent. The study period lasted from January 2015 (start of PIPAC program in our department) to January 2020. Patients in the PIPAC group were compared to all consecutive patients with laparoscopic staging alone (laparoscopy group)) for gastro oesophageal junction (GOJ) adenocarcinoma (Siewert II and III) classified uT3 or uT4 [12]. Baseline demographics were compared according to the age, gender, BMI (kg/m2), ASA score and Charlson Comorbidity Index (CCI) [13]. Staging laparoscopy with peritoneal washing was performed according to the current ESMO guidelines for all patients with resectable stage IB-III gastric adenocarcinoma, to exclude the presence of occult peritoneal carcinomatosis [14]. No prophylactic PIPAC was foreseen in this setting without suspected PSM on baseline imaging, and as a consequence no PIPAC could be delivered for patients with intraoperative diagnosis of peritoneal implants. Staging laparoscopy was uniformly performed in both groups, with systematic assessment of all abdominal regions according to Sugarbaker's peritoneal cancer index (PCI) [15]. All laparoscopies in both groups (laparoscopy alone and PIPAC) were performed before resection of primary. Criteria of exclusion was age < 18 years old and patients’ refusal to participate.

### Outcomes

Safety, tolerance and potential chemotherapy-related adverse events were assessed by documentation of postoperative complications according to the Clavien classification and by use of CTCAE v5.0 [16, 17]. Nausea and postoperative pain (at rest) were measured on routine basis by use of a visual analogue scale (0–10: maximal intensity) 3x/d.

### Data Management

Demographics, oncological and surgical data were retrieved from a prospectively maintained institutional database and entered in an a priori defined anonymized data base. The following variables were extracted: age, gender, primary tumour origin, body mass index, ASA class, Charslon Comorbidity Index (CCI) [13], intra-abdominal chemotherapy regimen (for PIPAC), PCI (Peritoneal Cancer Index), overall postoperative complications (according to Calvien-Dindo), postoperative pain and nausea (VAS- visual analog scale: 0–10). Analgesia protocols were comparable between the two groups without use of opioids only on demand.

### PIPAC procedure and safety considerations

PIPAC procedure has been detailed previously and was applied according to current recommendations and safety protocols [18]. Oxaliplatin was applied at a dose of 92 mg/m2 for carcinosis of colorectal origin. Cisplatin (7.5 mg/m2) in combination with Doxorubicin (1.5 mg/m2), with dose adaptation since 2019 according to Tempfer’s phase 1 trial (10.5 mg/m^2^ and 2.1 mg/m^2^) was used for ovarian, gastric, and other malignancies [19]. Aerosol chemotherapy was applied using electrostatic precipitation (ePIPAC) in our department since 2017 [20].

### Statistics and analysis

Continuous variables were presented as mean with standard deviation (SD) or median with interquartile range (IQR) according to their distribution. Categorical variables were reported as frequencies (%) and compared with chi-square test. Student t-test or Mann–Whitney test were used to compare continuous variables. A linear mixed-effect model to assess the effect of surgery type on VAS scores, when correcting for time. All statistical tests were two-sided and a level of 0.05 was used to indicate statistical significance. Statistical analyses were performed with GraphPad Prism 8 (GraphPad Software, Inc., La Jolla, CA, USA).

### Ethics

The study was approved by local Commission on Ethics in Human Research (CER-VD 2019–00747) and was conducted in compliance with the current version of the STROBE statement (www.strobe-statement.org) [21].

## Results

Forty-nine patients (M: F = 32: 17, mean age 60 ± 11 years) underwent during the study period either laparoscopy alone (LA) (n = 24) or laparoscopy + PIPAC (LP) (n = 25) group. LP group included 10 patients with colorectal primary (42%), 6 gastric (22%), 5 ovarian (20%) and 4 appendicular (16%). Median PCI in the LP group was 10 (Range: 0–22). Five patients (20%) in the LP had no macroscopic disease (PCI = 0), one of these five patients had positive cytology. No patient had concomitant IV chemotherapy during PIPAC procedure. All patients in the LA group had GOJ adenocarcinoma (16 uT3 (66%) and 8 uT4 (34%)), 3 of them had PC (13%) two with a PCI 3 one PCI 15 and one patient had positive cytology with a PCI of 0. Median surgical time (p = 0.001) and number of trocars were significantly different between the two groups (p = 0.011). There were no intraoperative complications in any of the 49 procedures. Baseline demographics and surgical details are displayed in Table 1.

**Table 1.** Staging laparoscopy alone (LA) *vs.* staging laparoscopy with PIPAC (LP): baseline demographics, surgical details

	Total n = 49	Laparoscopy alone n = 24	Laparoscopy + PIPAC n = 25	p-value
Demographics				
Gender (M: F)	32:17	20:4	12:13	**0.021**
Mean age (SD)	60.3 (10.7)	60.7 (10.9)	59.8 (10.7)	0.733
Mean BMI (kg/m2) (SD)	26.2 (5.4)	27.1 (6.1)	25.4 (4.7)	0.512
ASA score				0.873
1	1 (2%)	1 (4%)	-	
2	37 (76%)	17 (71%)	20 (80%)	
3	11 (22%)	6 (25%)	5 (20%)	
Median Charlson Comorbidity Index (IQR)	6 (4–7)	4 (4–6)	7 (6 -8)	0.012
Surgical details				
Median surgical time (min) (IQR)	77 (63–105)	64 (40–75)	99 (87–113)	**0.001**
Median n. of trocars (Range)	2 (2–4)	3 (2–4)	2 (2–3)	**0.011**
Port-a-Cath as additional procedures (n, %)	–	17 (71%)	–	–

Median (IQR- Interquartile Rang or Range), Mean (SD – Standard Deviation) or number (%) as appropriate. Statistical significance (p < 0 05) is highlighted in bold. ASA: American Association of Anesthesiologists physical status classification system. Charlson Comorbidity Index [13]

**Fig. 1.**
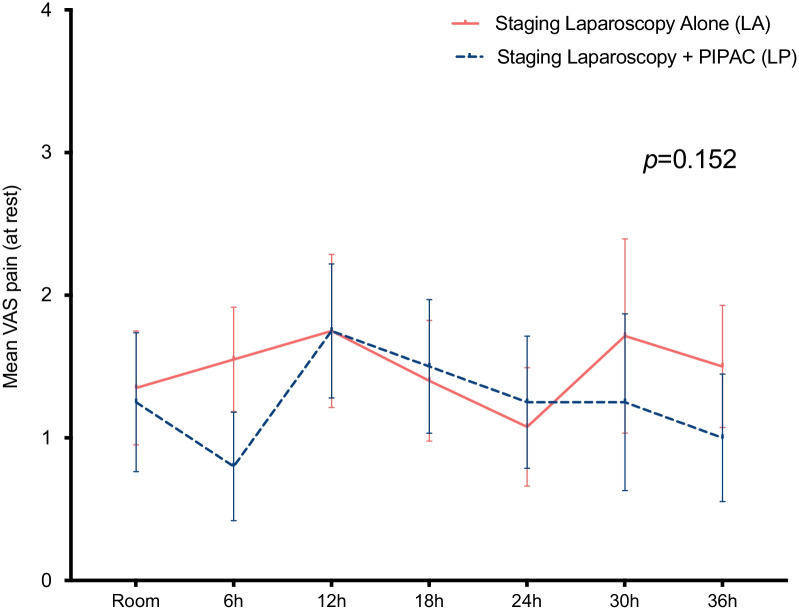
Staging laparoscopy alone (LA) *vs.* staging laparoscopy with PIPAC (LP): pain scores at rest. Evolution of pain scores over time after LA and LP procedure, at rest at different time points postoperatively. *VAS* visual analogue scale; *S.E.M.* standard error of the mean; Room: recovery room

There was no significant difference between LA *vs*. LP regarding length of stay, postoperative nausea and overall complications (Table 2). Post-operative complications were: 2 subcutaneous hematoma, 1 urinary retention, 1 ileus requiring nasogastric tube (NGT) in the LA group and 3 subcutaneous hematomas [1 requiring transfusion] and 1 urinary retention in the LP group. No postoperative complications were directly related to intra-peritoneal chemotherapy in the LP group. No difference was found between the two groups regarding post-operative pain. (Fig. 1).

**Table 2.** Staging laparoscopy alone (LA) *vs.* staging laparoscopy with PIPAC (LP): clinical outcomes

	Total n = 49	Laparoscopy alone n = 24	Laparoscopy + PIPAC n = 25	p-value
Overall complications (n, %)	8 (16%)	4 (16%)	4 (16%)	0.741
Grade I	4 (8%)	2 (8%)	2 (8%)	
Grade II	4 (8%)	2 (8%)	2 (8%)	
CTCAE (n, %)				
Grade I	4 (8%)	2 (8%)	2 (8%)	0.940
Grade II	2 (4%)	1 (4%)	1 (4%)	
Grade III	2 (4%)	1 (4%)	1 (4%)	
Median Length of Stay in days (IQR)	1 (1–3)	1 (1–2)	2 (1–3)	0.104

Median (IQR- Interquartile Rang or Range), Mean (SD – Standard Deviation) or number (%) as appropriate. Statistical significance (p < 0 05) is highlighted in bold. Complication according to Clavien-Dindo by use of CTCAE v5.0 [16, 17]

## Discussion

In this study, the combination of staging laparoscopy with PIPAC was equally safe and well tolerated as staging laparoscopy alone. Surgery time was longer in the LP group, but early clinical outcomes and hospital length of stay were similar. The potential benefits of added PIPAC remain yet to be investigated.

Preliminary published studies have shown good tolerance and safety of PIPAC [7, 8]. However, these studies have been carried out for the most part in palliative situations. This study investigates the effect of PIPAC in a neoadjuvant/prophylactic setting and aimed the tolerance of PIPAC to staging laparoscopy alone. The results of this preliminary study are encouraging and support further evaluation of PIPAC in a neoadjuvant setting.

PSM comprises a heterogeneous group of quite different primaries. Most frequent origins spreading within the abdominal cavity at initial presentation are ovarian (46%), oesogastric (14%), and colorectal tumors (5%) [1, 2, 4]. Staging laparoscopy for primary digestive malignancies allows identification of occult peritoneal disease [22, 23]. According to ESMO guidelines, staging laparoscopy is recommended for all patients with locally advanced gastro oesophageal adenocarcinoma (> cT3 and/or cN + stage) [14]. In particular, tumors that develop within the abdominal cavity (Siewert II and III) are more susceptible to present a peritoneal metastatic spread (6%–17%) upon initial diagnosis [24, 25]. Hence, diagnostic laparoscopy is an integral part of locally advanced gastric and gastroesophageal junction cancer staging [24]. Occult PSM precluding upfront curative surgery is discovered in 15 to 40% of patients with locally advanced gastric cancer during staging laparoscopy [26–28]. ESMO guidelines recommend a staging laparoscopy in all potentially resectable stage IB–III gastric tumors, [14] whereas the SAGES guidelines recommend staging laparoscopy for T3/T4 gastric cancer without evidence of lymph node or distant metastasis on high quality preoperative imaging [23]. In colorectal cancer, approximately 20–30% of patients with pT4 or perforated tumors develop metachronous peritoneal metastases, but staging laparoscopy is not performed systematically [29, 30]. In this situation, primary tumor resection is often necessary even in presence of PM to treat or even to prevent imminent obstruction, perforation or bleeding [23]; as such, the additional value of staging laparoscopy in the primary and metastatic colorectal cancer has not yet been clearly defined [31, 32]. However, 10% of occult peritoneal metastases for pT4 cancer are diagnosed during a planned second look laparoscopy 6 months after resection, even when no metastases are detected on high-resolution abdominal imaging [29]. For appendiceal tumors, the detection rate of PM is up to 23% at one year after primary resection for mucinous neoplasm. Thus, laparoscopy was suggested as a primary screening tool during postoperative follow-up, to identify occult metastases undetectable on CT scan [33]. In the context of ovarian epithelial cancer, staging laparoscopy was suggested as a routine in the initial diagnostic workup [34], leading to upstaging of the disease through detection of PM in 22.6% of patients [35].

Prognosis of patients with advanced PM is dismal and depends mainly on disease extent and response to therapy [7]. Resistance to systemic chemotherapy due to limited drug distribution in the peritoneum and limitations in early-stage disease diagnosis with non-invasive imaging make PM management particularly challenging [3].

Complete abdominal surgical exploration in high-risk patients with PM has been described and evaluated in prospective non-randomised studies [36–40]. Similarly, a systematic second look is proposed for early diagnosis of peritoneal metastases from colorectal origin, not visible on imaging. This second-look allows early treatment of patients with low PCI; although this may improve survival in selected cases, it rarely allows a curative treatment strategy. To overcome this problem intraabdominal chemotherapy was evaluated as adjuvant treatment in cohort studies [41–43]. These preliminary studies reported promising results of adjuvant HIPEC for high-risk colon cancer, decreasing the incidence of peritoneal metastasis to 0–4% after IntraPeritoneal treatment. Five randomized studies aimed to determine the efficacy of adjuvant HIPEC in patients with locally advanced colon cancer: PROPHYLOCHIP–PRODIGE 15 trial [44], COLOPEc trial [30], APEC Study [45], HIPECT4 trial [46] and PROMENADE trial (47). Systematic second-look surgery plus oxaliplatin-HIPEC did not improve disease-free survival compared to standard surveillance in ROPHYLOCHIP–PRODIGE 15 and COLOPEc, but was related to up to 41% of postoperative complications (grade 3–4) [30, 44]. HIPECT4 trial observed a reduced risk of peritoneal recurrence from 36 to 18% at 36 months for T4 colon-rectal carcinoma after adjuvant HIPEC [46]. Results of APEC and PROMENADE trials are still awaited [45, 47].

Pressurised intraperitoneal aerosol chemotherapy (PIPAC) has been proposed as an alternative mode for intraperitoneal drug delivery in certain situations, claiming improved distribution, enhanced tissue uptake, better tolerance, and repeatability using minimally invasive access [5, 6, 8]. Favourable initial reports [7] have triggered the adoption of PIPAC as a drug delivery technique. PIPAC has been proposed as an alternative method of intraperitoneal drug delivery, claiming improved distribution, enhanced tissue uptake, better tolerance and repeatability using minimally invasive access [5, 6, 8]. In recent systemic reviews, PIPAC is considered a safe and promising treatment alternative for patients with advanced isolated refractory peritoneal disease [7, 8].

Adjuvant PIPAC for high-risk patients is an intriguing concept which entails a risk of overtreatment. Added PIPAC in this study did not increase the risk or tolerance of staging laparosocopy alone. On the other hand, the risk of missed opportunities (no PIPAC with patients with positive cytology) is to consider and might favour local spread. Three prospective randomized trials are currently recruiting to assess PIPAC as adjuvant treatment: the GASPACCO [48] and PIPAC-OPC4 [49] trials for T3-4 Gastric Cancer and the PIPAC-OPC3 CC trial for high risk colon cancer [50].

Following the principles of the IDEAL framework allowing standardized approach, future prospective studies are needed to confirm the efficacy and oncologic benefits of PIPAC [51]. Furthermore, a registry for quality control supported by the International Society for the Study of Pleura and Peritoneum (ISSPP) was launched in 2020. This international database hosted at the University of Odense will facilitate future research with prospective monitoring [52].

The current study has some limitations which are mainly related to its retrospective nature and limited patient number and heterogeneity of groups. Differences between the comparative groups might have passed undetected due to type II error. Although baseline characteristics of patients were comparable there was no random allocation for the two groups with a consequent risk for selection bias. Arguably, patients in the PIPAC group might have been a higher risk for complications due to more advanced peritoneal disease (higher PCI) and prior treatments. This report could therefore be interpreted as indirect confirmation of the safety of PIPAC (i.e. same complication rates in "worse" patients). Even if the number of patients was low in both arms with heterogeneous patients, the groups were comparable for ASA score and only slightly different regarding the Charlson Comorbidity Index score. Furthermore, both comparative groups were treated by the same surgical team in the same hospital and with the same perioperative care strategies.

## Conclusions

In conclusion, neoadjuvant and prophylactic PIPAC could be proposed to patients undergoing staging laparosocopy for suspected or confirmed PSM with minimal increase in surgery time, but no increase in risk and tolerance of the procedure. Its oncological efficacy in this context is currently investigated under controlled conditions. Until then, PIPAC should only be performed in expert centers under standardized conditions and with and prospective monitoring and systematic patient follow-up.

## Data Availability

The datasets used and/or analysed during the current study are available from the corresponding author on reasonable request.

## Abbreviations

PIPAC: Pressurized IntraPeritoneal Aerosol Chemotherapy
PSM: Peritoneal surface malignancy
PCI: Peritoneal Cancer Index
PM: Peritoneal metastasis
